# Informing the design of a randomised controlled trial of an exercise-based programme for long term stroke survivors: lessons from a before-and-after case series study

**DOI:** 10.1186/1756-0500-6-324

**Published:** 2013-08-13

**Authors:** Leon Poltawski, Jacqueline Briggs, Anne Forster, Victoria A Goodwin, Martin James, Rod S Taylor, Sarah Dean

**Affiliations:** 1 University of Exeter Medical School, Veysey Building, Salmon Pool Lane, Exeter EX2 4SG, UK; 2 Stroke Research Network (Southwest), Royal Devon and Exeter Hospital, Barrack Road, Exeter EX2 5DW, UK; 3 Academic Unit of Elderly Care and Rehabilitation, Bradford Institute for Health Research, Bradford Teaching Hospitals NHS Foundation Trust/University of Leeds, 101 Clarendon Road, Leeds LS2 9LJ, UK; 4 Royal Devon and Exeter Hospital, Barrack Road, Exeter EX2 5DW, UK

**Keywords:** Stroke, Exercise, Feasibility study, Complex interventions

## Abstract

**Background:**

To inform the design of a randomised controlled trial (RCT) of an exercise-based programme for long term stroke survivors, we conducted a mixed methods before-and-after case series with assessment at three time points. We evaluated Action for Rehabilitation from Neurological Injury (ARNI), a personalised, functionally-focussed programme. It was delivered through 24 hours of one-to-one training by an Exercise Professional (EP), plus at least 2 hours weekly unsupervised exercise, over 12- 14 weeks. Assessment was by patient-rated questionnaires addressing function, physical activity, confidence, fatigue and health-related quality of life; objective assessment of gait quality and speed; qualitative individual interviews conducted with participants. Data were collected at baseline, 3 months and 6 months. Fidelity and acceptability was assessed by participant interviews, audit of participant and EP records, and observation of training.

**Findings:**

Four of six enrolled participants completed the exercise programme. Quantitative data demonstrated little change across the sample, but marked changes on some measures for some individuals. Qualitative interviews suggested that small benefits in physical outcomes could be of great psychological significance to participants. Participant-reported fatigue levels commonly increased, and non-completers said they found the programme too demanding. Most key components of the intervention were delivered, but there were several potentially important departures from intervention fidelity.

**Discussion:**

The study provided data and experience that are helping to inform the design of an RCT of this intervention. It suggested the need for a broader recruitment strategy; indicated areas that could be explored in more depth in the qualitative component of the trial; and highlighted issues that should be addressed to enhance and evaluate fidelity, particularly in the preparation and monitoring of intervention providers. The experience illustrates the value of even small sample before-and-after studies in the development of trials of complex interventions.

## Findings

### Introduction

The personal and economic costs of disability following stroke are considerable, and it is recognised that appropriate longer term interventions are required to reduce them [1-3]. This has led to the development of long-term community-based provision for stroke survivors, both to address the sequelae of stroke and to encourage and support self-management by the individual. In the realm of physical rehabilitation, much service provision has been governed by a presumption of diminishing returns – that input by practitioners much later than six months post-stroke is unlikely to result in substantial improvement for the individual [4]. This assumption is being challenged [3,4]. However, systematic reviews of trials specifically addressing the rehabilitation of long-term stroke survivors have highlighted the scarcity of high quality studies, and concluded that further evidence is required to judge the types of intervention, and the intensity levels, that might benefit this group [5-7].

In several countries, health care providers and local governments are beginning to set up a range of community-based exercise programmes [8-12]. Although individual elements of such programmes may be evidence-based, little data are available to support particular “packages”. This is partly because multiple elements , including personal and environmental factors, may interact to produce a range of outcomes. These programmes therefore constitute complex interventions and should be evaluated as such [13]. One such intervention is Action for Rehabilitation from Neurological Injury (ARNI: http://www.arni.uk.com), a programme that has been specifically developed for stroke survivors with a wide range of disability levels. ARNI gives particular emphasis to functional strengthening, and includes learning compensatory strategies for specific functional tasks such as independently getting off the floor and avoiding collisions in crowds. Programmes are based on negotiated goals, and involve controlled risk-taking (e.g. rapid whole-body movements) to build confidence, along with self-monitoring and shared problem-solving, to promote exercise self-efficacy and self-management. ARNI was designed as a one-to-one intervention, delivered primarily by Exercise Professionals after clinical rehabilitation has ended, and has been adapted for individual and group classes by a number of providers in the UK.

Guidance on the development and evaluation of complex interventions recommends that, prior to conducting randomised controlled trials of such interventions, studies should first be undertaken to address issues such as acceptability of interventions to patients, and trial design [12,13]. The reporting of such exploratory studies may provide valuable insights for broader research efforts. In preparation for a randomised controlled trial of the ARNI intervention for long-term stroke survivors, we conducted several related studies to inform its development, including focus groups, a survey of current programmes, and a case series study of the intervention. This paper describes the case series study, whose objectives were to examine the acceptability of the ARNI programme to participants, to identify issues of feasibility and fidelity assessment that should be addressed in a trial of the intervention, and to evaluate a variety of outcome measures that might be used in a trial.

## Methods

A longitudinal case series design was used: long-term stroke survivors were assessed using mixed methods before, during and after participating in an exercise programme, which comprised one-to-one training sessions led by an ARNI-qualified Exercise Professional. The assessment included baseline data collection, subjective and objective outcome measurement at baseline, three and six months, and qualitative 1:1 interviews with participants and trainers conducted before and after the intervention. Additional process data was gathered through observation of training sessions and the use of diaries and training records. This study design was chosen for its capacity to describe and explore the intervention, and to investigate how it is implemented and received [14]. In particular, case-by-case analysis of both quantitative and qualitative data gathered at multiple time points can aid the identification of factors that influence individual experiences, choices and outcomes in a complex intervention [15]. The study was approved by the local committee of the UK NHS Research Ethics Service (Reference 11/H0206/6).

### Participants

Eligible participants had their stroke at least 6 months prior to enrolment in the study, were discharged from existing NHS rehabilitation, had a modified Rankin score (mRS) [16] of between 2 and 4, were capable of consenting to and participating in a physical exercise programme, and had no contraindications to moderate exercise. A screening questionnaire regarding cautions and contraindications to exercise, based upon expert guidelines [17], was completed by each participant’s GP prior to enrolment in the study. Several local recruitment routes were used: referral by rehabilitation clinicians, recommendation by EPs, promotion in service user groups and meetings, and study advertisement in the regional Stroke Research Network newsletter. Those continuing to express an interest after seeing the Participant Information Leaflet and telephone screening were visited at home to discuss expectations and programme requirements before giving consent. Six participants were recruited, the number being determined pragmatically on the basis of available resources.

### Intervention

The ARNI programme was provided by two Exercise Professionals (EPs), each with over five years’ experience as personal fitness trainers and qualified to an advanced level on a UK register of Exercise Professionals . Prior to involvement in the study, neither had experience of working with stroke survivors. They were prepared to deliver the intervention by accreditation as ARNI Trainers following 5 days of training, passing theoretical and practical examinations, and submitting a case study after an assessment and several sessions working with a stroke survivor. The training includes information about stroke and its consequences, and teaches ARNI programme principles and a repertoire of functionally-oriented exercises and compensatory strategies. A suggested programme structure is provided, but the trainer is expected to develop a personalised programme for each participant, based upon negotiated goals. Trainers are provided with a comprehensive manual describing all the exercises developed for the programme [18]. Table 1 lists the main ARNI principles, identified by the investigators after analysis of programme documentation and discussion with the originator of the ARNI programme.

**Table 1:** ARNI training principles

• Functional focus	• Supervised risk-taking / boundary-pushing
• Strengthening for function	• Promoting self-management of exercise programme
• Encourage use of affected limbs	• Personalisation of training programme
• Sustained working at demanding levels	• Promote commitment to regular exercise

The ARNI programme is designed to be progressive and ongoing, and does not have a prescribed endpoint. For the purposes of this study, it was conducted over 12–14 weeks, during which the participant was to receive 24 hours of one-to-one training, typically conducted as hourly sessions twice weekly, either at the participant’s home or in a gym. In addition, participants were expected to undertake at least 2 hours of unsupervised exercise each week, practising ARNI exercises and engaging in other forms of physical exercise, such as swimming, walking or during attendance at a local gym.

### Data collection

Baseline assessment included data collection on participant demographics, lifestyle and medical history, and previous post-stroke rehabilitation experience. A range of outcomes were measured subjectively using patient-rated questionnaires, and functional mobility was assessed objectively by a researcher using the Performance Oriented Mobility Assessment (POMA - Gait and Balance) [19] and the Timed Up and Go (TUG) test [20] (see Table 2). Assessments were conducted at baseline, immediately after the programme and three months later (i.e. three and six months post-baseline respectively).

**Table 2:** Quantitative outcome measures employed in study

**Instrument**	**Measures participant perception of**
Nottingham Extended Activities of Daily Living (NEADL) scale [21]	Level of independence completing everyday tasks
Stroke Self-Efficacy questionnaire (SSEQ) [22]	Confidence in own ability to complete everyday tasks
Fatigue Assessment Scale (FAS) [23,24]	Fatigue in daily life
Reintegration into Normal Living Index (RNLI) [25]	Limitations in taking on life and social roles
EQ5D [26]	Health-related quality of life
SF36 [27]	Health-related quality of life (physical & mental health sub-scales)
Performance oriented mobility assessment [19]	Gait quality and balance
Timed up and go test [20]	Functional mobility

Semi-structured qualitative individual interviews were also conducted with each participants (together with their family partner where available) at the same time points. The interviews addressed programme content and process, participant experience and satisfaction, benefits and adverse incidents. Issues arising at first interview (e.g. their expectations and concerns) were returned to in subsequent interviews. Interviews were electronically recorded and transcribed verbatim for subsequent analysis. Participants were also asked to keep a diary recording any significant events, positive or negative, associated with their involvement in the programme.

Fidelity to ARNI programme principles and requirement by EPs and participants was assessed by several mechanisms: EPs were asked to record assessments, the content of each training session, progress and adverse events, and any suggested home-based exercises; the participant diaries include brief sections to summarise any physical exercise done outside the training sessions; and three training sessions for each participant – occurring near the beginning, middle and end of the programme – were observed by a single researcher and their content analysed using a pro-forma developed for the purpose.

### Data presentation and analysis

Because of the small sample size, inferential statistical tests of quantitative outcomes were not conducted. Instead, quantitative outcomes were represented graphically at each time point for each participant. Qualitative data was analysed at both group and individual levels. A general inductive approach was used for group level thematic analysis [28]. Two researchers independently read all the participant interview transcripts, identified text segments relating to the research aims, and iteratively organised the segments under a set of themes and sub-themes considered to capture the most significant issues emerging from the data at each time point. The researchers compared their findings and agreed a set of pertinent themes. In addition, qualitative data available from other sources, including personal histories and demographics, diaries and records of exercises, were considered on a case-by-case basis to provide additional insights relating to intervention outcomes and to themes emerging from the participant interview analysis.

### Recruitment and screening

**Figure 1:**
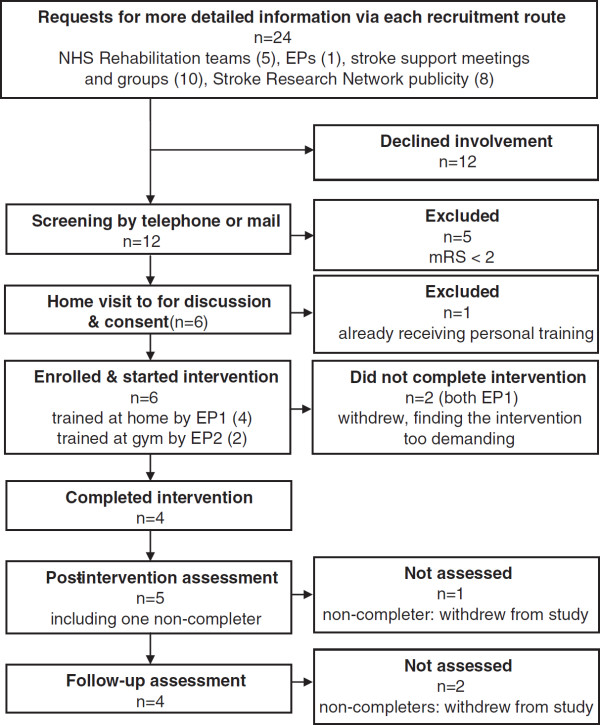
Recruitment, participation and assessment.

Initially the intention was to recruit exclusively through local rehabilitation health professionals, but low numbers of referrals led to additional routes being explored, as listed in Figure 1.

### Participant characteristics

Six people participated in the study, five male and one female. At baseline, their mean age was 67 years (range 57–72 years) and mean time since stroke was 8.5 years (range 1–16.5 years). The median modified Rankin Score was 2.5 (range 2–4). Three reported exercising with moderate exertion at least once a week.

### Programme participation and intervention fidelity

Individual training began between July and September 2011. Four participants were seen at home by one Exercise Professional (EP1) and two at a private gym by another (EP2). The location was decided according to participant’s access to transport. Four completed the programme, receiving 22–24 sessions over 12–13 weeks. Two, both trained at home by EP1, left the programme early, one (CS1) after four sessions, the other (CS4) after nine.

The fidelity checking procedures suggested that training had been tailored to the capacity of the individuals, and generally conducted at an appropriately demanding level. Use of the specific task-related exercises specified in the ARNI manual was variable, with training often focussed more on strengthening and overall fitness. Both EPs encouraged strengthening of the stroke-affected limbs, but only EP1 gave consistent attention to upper limb function. Goals were discussed in early sessions but it was not evident that they were revisited at later sessions. Attention was given to developing knowledge of exercise types and understanding of exercise principles. Home exercises were suggested, though not consistently checked by the EPs.

Examination of written records and post programme interviews suggested that three participants exercised regularly between supervised sessions, but only one completed their log consistently. Thus it was not possible to obtain a reliable estimate of time spent in independent exercise.

### Quantitative outcomes

**Figure 2:**
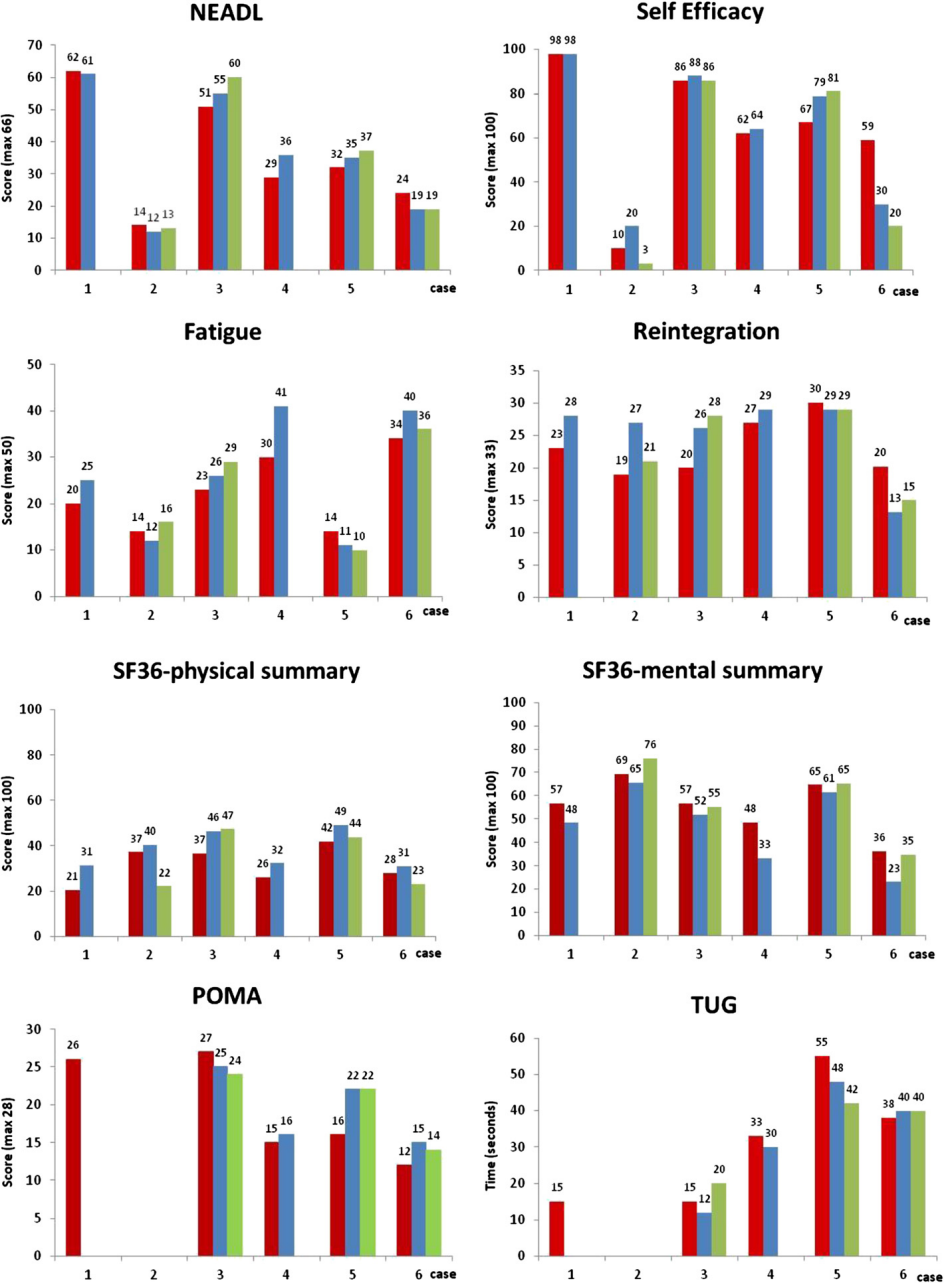
**Outcomes for cases 1 – 6 at baseline (red square symbol), post-programme (blue square symbol) and 3 month follow-up (green square symbol).** An increase in score represents an improvement in outcome, except for the Timed Up and Go (TUG) test and the Fatigue Assessment Scale (FAS), where improvements are indicated by a lower score.

Individual outcome measure scores at each of the three time points are shown in Figure 2. POMA and TUG tests were not conducted for CS2, who was non-ambulant. Other missing data was due to study withdrawal. The quantitative data demonstrated little change across the sample, but marked changes on some measures for some individuals. No individuals showed consistent change across the measures, although two (CS3 and CS5) improved on a majority of them and one (CS6) deteriorated on a majority. Marked and sustained improvements were observed in gait quality and speed for only one person, and moderate improvements for two others, but some deterioration was evident in one case. No adverse events were reported by either participants or EPs.

### Qualitative interviews

All completers were interviewed at all time points. Of the non-completers, one consented to a post-programme interview but the other preferred only a brief telephone discussion. Follow-up interviews were not conducted in either case. A number of themes relating to outcomes were identified in the analysis of interviews:

1. **Individual physical benefits.** All completers reported a range of physical improvements. These included increased upper limb and core strength, greater range and control of upper limb movement, better balance and overall fitness. Improvements in functional capacities and activities included ability to transfer, being able to turn over in bed, better walking quality, and easier lifting and manipulation of kitchen equipment.

2. **Small changes and their personal significance.** Benefits that might have been missed or seen as minor by others, were reported as personally important by the individual. For instance, one person was delighted to be able to move his fingers on a previously inert hand, even though this did not produce any functional improvement. Another felt more in control and comfortable after gaining sufficient core strength to stay upright in a cornering vehicle. The significance of such benefits appeared to rest on their psychological value to the individual, which was apparent in several dimensions, described below.

3. **Awakening to personal potential.** All participants were long term stroke survivors who had been discharged from clinical rehabilitation with the message that little further improvement was likely. Participation in the programme convinced the completers, sometimes to their surprise, that they were capable of further progress. This was said to engender hope, help build self-esteem, self-belief and a sense of continuing on an improving trajectory. However, confidence may have been undermined for those who did not complete the programme. One spoke of frustration and disappointment on not being able to cope with its demands.

4. **Self-management and support.** Completion of the programme was reported to enhance self-management capacity by increasing the personal repertoire of exercises and activities, and knowledge of training principles and their application after stroke. However, intentions to self-develop exercise programmes that were expressed just after the programme were often not carried through by follow-up interview: there was an admission that motivation was hard to sustain once supervision had ended, and one completer reported working hard within sessions but doing virtually no exercise outside them.

Several issues relating to programme delivery were also identified in the analysis:

(i) The high demand, intensive approach was seen as key to becoming aware of personal potential by those who completed the programme. However it was also a source of fatigue, and was cited as a reason for leaving the programme by those who withdrew.

(ii) Whilst health professionals were often felt to be risk-averse and having low expectations of their patients, the high expectation, can-do attitude of the EPs was valued and cited as a strong motivational factor by the completers. Conversely, it was seen as sometimes unrealistic by the non-completers.

(iii)  For several participants, being treated as a client to be coached and trained, rather than a patient to be made well, helped create a sense of normalisation which contributed to self-confidence and self-image.

### Reasons for non-completion

The primary reason given by the two participants who did not complete the programme was an inability to cope with its demands. Both felt that the EP had unreasonable expectations of what they could achieve, and CS1 complained of substantial post-exercise soreness. Other factors may also have been involved. CS1 had a range of co-morbidities and CS4 identified increasing levels of fatigue that preceded enrolment in the study. Neither reported exercising regularly before the programme, and – in contrast to the completers – neither lived with a partner who supported their involvement in the programme. They were also the first two participants seen by EP1, so it may also be that the EP was insufficiently experienced to judge the appropriate intensity level.

## Discussion

This study raised a number of issues that will inform the development of the protocol for a RCT of the ARNI programme. These relate to the recruitment and selection of participants, ways of enhancing and assessing fidelity to the intervention, focusing on methods of sustaining commitment and benefits, and appropriate choice and interpretation of outcome measures.

### Recruitment and selection

The decision to extend recruitment from sole dependence on clinical referrals to the use of multiple routes, was vindicated by the fact that participants were drawn from all of the routes. Clinicians may initially be reluctant to refer patients to novel services in their early stages of development [29]. In any case, the majority of participants in this programme and many of those registering an interest in it had long been discharged from therapy services. Thus, recruitment from additional routes may be desirable to reach a broader population of longer term stroke survivors. Half of those requesting information about the trial did not indicate further interest; they may have felt they did not satisfy the eligibility criteria or been put off by the information, although this had been previously been assessed by members of the study service user advisory group. Study recruitment may be affected by promotional materials [30] and following this study we addressed this issue through further consultation with our service user group and focus groups.

The two non-completers in this study, and the completer with least evidence of benefit, reported very low levels of exercise before joining the programme. Obesity, co-morbidity, socio-economic status and the lack of a supportive partner may also have modified outcomes in some cases. Their potential influence could be tracked through subgroup analysis in a RCT. For instance, pre-intervention exercise behaviour can be measured using a variety of instruments [31], and could be used for stratification in a large trial. There was no evidence that mRS score influenced outcomes, so those with more severe impairments need not be excluded from a trial. This is valuable because exercise trials for longer term stroke survivors often exclude those with more severe impairments [5,6].

### Intervention fidelity

Observing sessions and rating the application of ARNI training principles proved an effective method of assessing fidelity to the intervention. Although only selected sessions were observed, triangulating the findings with the examination of EP notes enabled a picture of training content, process and progression to be developed. A more detailed report on this process is in preparation. The analysis suggested that adherence was only partial in a number of areas, and that greater focus on the ARNI principles is required in the briefing of EPs delivering the intervention. In a trial, fidelity-checking using this approach with a sub-sample of participants for each EP is feasible.

As is common in self-report of exercise behaviour, few data were available for analysis. The ARNI programme aims to increase self-directed exercise behaviour, and its benefits are predicated on several hours of exercise each week [18], not all of which can feasibly be supervised. Hence the objective measurement of physical activity in a trial of this intervention should be considered, for example by the use of accelerometry.

Higher activity levels were not sustained after the programme, and completers expressed a need for ongoing support to maintain their commitment. A greater focus on goal setting and progress-reviewing skills during the programme may be helpful in this regard. Provision of tailored advice and sign-posting to local services and facilities near the end of the programme, or periodic follow-up training sessions to coach and advise on progression may also be beneficial. These options were recommended for exploration in development of the subsequent trial protocol.

### Outcomes

Measuring the effects of the intervention using quantitative instruments was problematic because data regarding their psychometric properties, particularly responsiveness to change, is limited. The minimum significant difference (MSD) is the smallest score change that is unlikely to be due to random error alone. MSD estimates of five points for individuals have been suggested for the Reintegration into Normal Living Index (RNLI) [32] and the POMA [33]. By these criteria, three of the participants improved and one deteriorated on RNLI, and one improved in POMA score. Of greater importance is the minimum clinically important difference (MCID), which is the smallest change that would be considered worthwhile from a clinical point of view. An MCID of five points has been suggested for the total NEADL score [21], and three participants saw such improvement. It has been suggested that changes of up to 40 points in individual SF36 scores may be required to be clinically significant [34]. No such changes were recorded so the quantitative measures provide only limited evidence of meaningful change.

In contrast, qualitative accounts suggested that all those completing the programme derived considerable personal benefit from participation. It may be that specific changes reported were not addressed by the measures, or may have been obscured by the lack of change in other areas assessed by each instrument. No standardised instrument can address all potential benefits, but this can lead to underestimates of effects if the individual experiences a change as personally significant. Personalised instruments such as Goal Attainment Scaling [35] are available, although patients can find initial goal setting for interventions problematic [36]. In any case, in this study the benefits were often unanticipated and their significance only became apparent in hindsight.

Other studies of exercise after stroke have identified mismatches between quantitative and qualitative data [37,38]. In a trial of a community-based exercise and education scheme, qualitative accounts suggested a variety of benefits that were not revealed by quantitative measures, including facilitating the creation of a positive post-stroke identity and empowerment to self-management through knowledge acquisition [39]. Along with our own findings, such studies underline the need to interpret quantitative findings in the light of qualitative accounts. Alternative quantitative measures could be considered for use in a RCT, including those addressing psychological variables such as self-concept and hope.

This study had a number of limitations. The small sample size and use of outcome measures lacking established MSD values meant that conclusions about changes at the sample group level, and about the utility of these particular outcome measures, could not definitively be drawn. The broad eligibility criteria resulted in a diverse sample, so that explanations for non-completion and variations in outcome are speculative, and require testing before being used to inform a full trial protocol. The use of EPs relatively inexperienced with this population and in delivering the ARNI programme may have reduced the impact of the intervention, particularly in improving functional outcomes. Nevertheless, the study was valuable in generating data and experience to inform the next phase of our research. Several of these are listed in Table 3.

**Table 3:** Recommendations arising from this study to inform development of an RCT

1.	Review recruitment strategy, particularly the variety of recruitment routes and the content of promotional materials.
2.	Include in the protocol a plan to investigate reasons for any withdrawals from study and implications for eligibility criteria and/or intervention design.
3.	Enhance intervention fidelity through additional briefing and formative evaluation of trainers, to ensure a focus on intervention essentials.
4.	Consider use of objective measures of exercise behaviour, including accelerometry, in evaluation of intervention process and outcomes.
5.	Investigate the range of psychological impacts of participation through quantitative outcome measures and qualitative enquiry.

## Conclusions

In accordance with Medical Research Council’s recommendations for the evaluation of complex interventions, we undertook a case series study to inform the design of a randomised controlled trial of an ARNI exercise programme for long-term stroke survivors. The study provided important data and experience, which generated a number of recommendations for the trial conduct and design. It confirms the value of conducting small exploratory studies, even with limited resources, as part of the work-up for full evaluative RCT.

## Abbreviations

ARNI: Action for rehabilitation from neurological injury; CS: Case; EP: Exercise professional; mRS: Modified rankin score; FAS: Fatigue assessment scale; NEADL: Nottingham extended activities of daily living; MCID: Minimum clinically important difference; MSD: Minimum significant difference; POMA: Performance oriented mobility assessment; RCT: Randomised controlled trial; RNLI: Reintegration into normal living index; TUG: Timed up and go.

## Competing interests

The authors declare that they have no competing interests.

## Authors’ contributions

LP was lead author and conducted day to day management of the study; SD was principal investigator of the study and contributed to data analysis. JB, AF, MJ, VG, RT and SD were involved in design of the study and contributed to the writing or review of this manuscript. All authors approved the final version of the manuscript.

## Authors’ information

LP is a research fellow; JB is manager of the Stroke Research Network (Southwest); AF is Professor of Stroke Rehabilitation, Bradford Teaching Hospitals NHS Foundation Trust/University of Leeds; VG is a Senior Research Fellow and physiotherapist; MJ is Consultant Stroke Physician/Honorary Associate Professor at the Royal Devon & Exeter Hospital/University of Exeter Medical School; SD is a Senior Lecturer in Health Services Research.
